# Increased expression of sialic acid in cervical biopsies with squamous intraepithelial lesions

**DOI:** 10.1186/1746-1596-5-74

**Published:** 2010-11-22

**Authors:** Dolores López-Morales, Julio Reyes-Leyva, Gerardo Santos-López, Edgar Zenteno, Verónica Vallejo-Ruiz

**Affiliations:** 1 Laboratorio de Biología Molecular y Virología, Centro de Investigación Biomédica de Oriente, Instituto Mexicano del Seguro Social, Km 4.5 Carretera Federal Atlixco-Metepec, C.P. 74360 Metepec, Puebla, México; 2 Laboratorio de Inmunología, Departamento de Bioquímica, Facultad de Medicina, UNAM, 04510, México

## Abstract

**Background:**

Altered sialylation has been observed during oncogenic transformation. Sialylated oligosaccharides of glycoproteins and glycolipids have been implicated in tumor progression and metastases. In the cervical cancer high levels of sialic acid have been reported in the patients serum, and an increased of total sialic acid concentration has been reported for the cervical neoplasia and cervical cancer. This study investigates the changes in expression and distribution of α2,3-linked sialic acid and α2,6- linked sialic acid in low and high squamous intraepithelial lesions and in normal tissue.

**Methods:**

Lectin histochemistry was used to examine the expression and distribution of sialic acid in different grades of cervical neoplasia. We applied *Maackia amurensis *lectin, which interacts with α2,3-linked sialic acid and *Sambucus nigra *lectin specific for α2,6-linked sialic acid.

**Results:**

The histochemical analysis showed that α2,3-linked sialic acid and α2,6- linked sialic acid increased in intensity and distribution in concordance with the grade of squamous intraepithelial lesion (SIL). These results are in concordance with a previous study that reports increased RNAm levels of three sialyltransferases.

**Conclusions:**

These results show that the change in sialylation occurs before cancer development and may play an important role in cellular transformation. These findings provide the basis for more detailed studies of the possible role of cell surface glycoconjugates bearing sialic acid in the cellular cervix transformation.

## Background

It is well known that tumorigenesis and metastasis are frequently associated with altered structure and expression of oligosaccharides on cell surface glycoproteins and glycolipids [1-6]. Sialylated glycoconjugates expression has been shown to change during development, differentiation, and disease and oncogenic transformation. A general increase in sialylation of cell surface glycoconjugates of carcinoma cells has been detected [7]. These changes in sialylation are related to invasion and metastasis [8-11].

Different studies in serum from patients with cervical cancer showed that sialic acid is elevated even in the early stages of carcinoma, and levels of these glycans decreased to near normal levels after radiotherapy [12,13]. Total sialic acid concentration of cervical tissue has been analyzed. Results showed a slight elevation in benign inflammatory lesions, moderate elevation in severe dysplasia and preinvasive carcinoma and high elevation in invasive cervical carcinoma [14]. Sialic acids are widely distributed in nature as terminal sugars of oligosaccharides attached to proteins or lipids. Sialic acids are linked to galactose via α2,3 or α2,6-linkage or linked via α2,6-linkage to galactosamine or N-acetylgalactosamine. Sialic acids may be linked to the C8 position of another sialic acid residue [15]. The biosynthesis of sialylated oligosaccharide sequences is catalyzed by a family of enzymes named sialyltransferases [15]. A correlation between sialic acid and sialyltransferase expression has been detected [16]. Altered expression of sialyltransferase in different cancers is well documented [17-20]. Increased expression of ST3Gal III and ST6Gal I was correlated with poor prognosis and lymph node metastasis in cervical cancer [21,22] In a previous study, our group investigated the sialyltransferase expression in cervical premalignant lesions and normal tissue. Increased expression of ST3Gal III, ST3Gal IV and ST6Gal I correlated with the grade of squamous intraepithelial lesion (SIL) [23].

In the present study we have performed lectin histochemistry in order to determine the level of expression and the distribution of α2,3 and α2,6 sialic acid in normal tissue and in low- and high-grade cervical intraepithelial lesions in order to determine the changes in sialylation at early stages of transformation in cervical neoplasia.

## Materials and methods

### Reagents

*Sambucus nigra *(SNA specific for NeuAcα2,6Gal-GalNAc); *Maackia amurensis *(MAA, specific for NeuAcα2,3Gal); and agglutinins biotinylated were purchased from Vector (Burlingame, CA, USA) and used at a concentration of 5 μg/ml. Signal amplification was realized with the TSA biotin system kit from NEN Life Science (Boston, MA, USA).

### Tissues

A retrospective study was carried out using material from the tissue collection at the Clinics of Dysplasia, Hospital General Regional No. 36 and Hospital General de Zona No. 5, Metepec, Mexican Institute of Social Security. Samples of normal cervix were obtained from uterus of patients who had undergone total hysterectomy due to uterine myoma. The biopsies were fixed in 3% phosphate-buffered formaldehyde and embedded in paraffin according to a standard protocol [24]. All samples were obtained according to the guidelines of the Human Ethics Committee of our Institution. This study involved 23 specimens classified by a pathologist according to the Bethesda System in normal, low SIL (LSIL) or high SIL (HSIL).

### Lectin Histochemistry

Paraffin sections (5 μm) were cut and placed on poly-L-lysine-coated glass slides. The sections were deparaffinized following published methods [24]. Sections were incubated in methanol containing 0.3% H_2_O_2 _for 30 min to block the activity of endogenous peroxidase. Hereafter, every step was followed by washing three times with 0.1 M Tris-HCl (pH 7.5), 150 mM NaCl. Slides were incubated with blocking buffer (0.1 M Tris-HCl, pH 7.5, 0.15 M NaCl, and 0.5% blocking reagent (supplied in kit) for 30 min at room temperature. Sections were incubated with 5 μg/ml biotin-conjugated lectins SNA and MAA for 1 h at room temperature. After this, sections were incubated with horseradish peroxidase-conjugated streptavidin for 30 min at room temperature. Biotin signal amplification was performed for 10 min at room temperature using biotinylated tyramide. Sections were incubated one more time with horseradish peroxidase-conjugated streptavidin for 30 min. Colored reaction product was obtained with 0.01% 3,3-diaminobenzidine tetrahydrochloride.

To confirm lectin specificity, enzymatic desialylation was performed by incubating tissue sections with neuraminidase from *C. perfringes *(50 mU/ml in 50 mM citrate pH 6.0, 0.9% NaCl, 0.1% CaCl_2_) for 30 min at 37°C. Negative controls of the technique were performed in samples processed without lectin. Sections were observed in an Olympus microscope (Olympus, Tokyo, Japan), and the images were processed with the Metamorph program (Universal Imaging Co., Downingtown, PA, USA).

The intensity of carbohydrate expression was semiquantitatively classified into five groups on the basis of the intensity of positive cells: 0, negative; 1, 2, 3 and 4 (very low, low, middle and high expression) and was correlated with histological diagnosis. We also evaluated the distribution of expression in the different epithelium layers. The expression was detected in the basal, parabasal, intermediate and superficial layers in order to be able to differentiate the layers or percentage of the epithelium when we could not differentiate the layers.

## Results

In the present study we analyzed a total of 23 biopsies: 9 cases of LSIL, 9 cases of HSIL and 5 biopsies with normal epithelium.

### Sialic Acid Expression

In order to determine the change in expression of α2,3 and α2,6 linkage sialic acid related to neoplastic transformation of the cervical epithelium, we performed lectin histochemistry assays with MAA and SNA.

#### α2,3 Sialic Acid Expression

Four out of five normal biopsies showed expression of α2,3 sialic acid. The higher expression was detected in the basal layer and only two samples with very low expression were detected in the medium and superficial layers (Table 1).

**Table 1:** α2,3 Sialic acid expression and distribution in samples with normal, LSIL, and HSIL

Normal			
**# sample**	**Basal**	**Intermediate**	**Superficial**

15	3	0	1

16	1	0	0

26	2	1	1

30	0	0	0

34	4	1	0

Mean intensity	2	0.4	0.4

			

**LSIL**			

11	3	0	0

13	2	0	0

21	2	0	0

22	3	0	0

35	2	2	2

38	3	2	1

41	3	0	1

43	2	1	2

48	3	1	1

Mean intensity	2.6	0.4	0.8

			

**HSIL**			

17	1	2	2

18	1	2	1

20	2	1	1

23	3	0	0

24	2	1	1

33	2	0	0

39	3	2	2

49	4	1	1

Mean intensity	2.2	1.1	1.0

HSIL, high-grade squamous intraepithelial lesion; LSIL, low-grade squamous intraepithelial lesion. Lectin binding was evaluated as follows: 0, absent; 1, very low intensity; 2, low intensity; 3, medium intensity and 4, high intensity. The reaction intensity was evaluated in the different layers of the epithelium (Basal, Intermediate and Superficial).

In all samples from the group with LSIL, sialic acid in α2,3 linkage was detected. They showed low and medium expression in the basal layer. For the intermediate and superficial layers, the expression was restricted to three and five samples, respectively with very low or low expression (Table 1).

**Figure 1:**
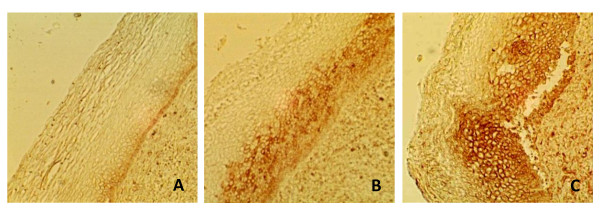
**α2,3 Sialic acid expression in cervical samples with normal (A), LSIL (B) and HSIL (C) diagnosis**. Sialic acid in α2,3 linkage was recognized with biotinylated MAA lectin. Amplification 100×.

Sialic acid expression in α2,3 linkage was detected in all HSIL samples analyzed (8/8) with a variable intensity of expression in the basal cells (1-4). The expression increased in the medium and superficial layers that extended in most cases to 66% or the total of the epithelium. In the layers with loss of cellular differentiation, the reaction intensity was higher with respect to layers with cellular differentiation (Table 1). The general pattern of α2,3 sialic acid expression showed an increased expression and distribution in the samples with SIL with respect to normal epithelium (Figure 1).

#### α2,6 Sialic Acid Expression

α2,6 Sialic acid was detected in all normal samples (5/5). Only one sample showed expression in the basal layer. All samples showed expression in the medium layers with variable intensity of expression. Four out of five samples showed expression in the superficial layers (Table 2).

**Table 2:** α2,6 Sialic acid expression and distribution in samples with normal, LSIL, and HSIL

Normal			
**# sample**	**Basal**	**Intermediate**	**Superficial**

15	0	3	3

16	0	1	0

26	0	2	1

30	0	1	1

34	3	3	2

Mean intensity	0.6	2.0	1.4

			

**LSIL**			

11	0	0	3

13	0	2	2

21	0	0	3

22	0	0	1

35	2	2	2

38	1	1	1

41	2	1	3

43	0	1	2

48	1	0	1

Mean intensity	0.7	0.8	2.0

			

**HSIL**			

1	3	2	1

17	2	3	3

18	1	4	3

20	1	3	3

23	0	3	3

24	3	3	3

33	3	2	2

39	0	2	3

49	3	1	2

Mean intensity	1.8	2.6	2.6

HSIL, high-grade squamous intraepithelial lesion; LSIL, low-grade squamous intraepithelial lesion. Lectin binding was evaluated as follows: 0, absent; 1, very low intensity; 2, low intensity; 3, medium intensity and 4, high intensity. The reaction intensity was evaluated in the different layers of the epithelium (Basal, Intermediate and Superficial)

In all LSIL samples, α2,6 sialic acid was detected. It was distributed in the intermediate and superficial layers. Expression at the basal and intermediate layers was very low and low, with increased expression in the superficial layer (Table 2).

All samples with HSIL diagnosis showed a medium expression of α2,6 sialic acid, with a distribution in all of them in the intermediate and superficial layer (9/9). In only two samples, expression in the basal cells was not detected (Table 2).

**Figure 2:**
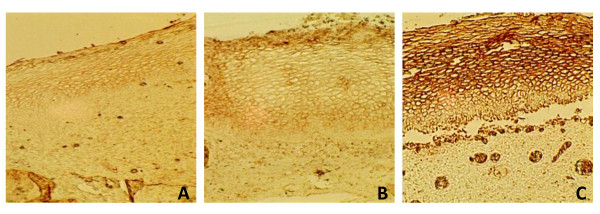
**α2,6 Sialic acid expression in cervical samples with normal (A), LSIL (B) and HSIL (C) diagnosis**. Sialic acid in α2,6 linkage was recognized with biotinylated SNA lectin. Amplification 100×.

The general pattern of α2,6 sialic acid expression showed an increase in intensity of expression and distribution in HSIL samples. α2,6 Sialic acid expression was not detected in basal cells, with one exception (Figure 2).

## Discussion

Glycoconjugates on the cell surface play important roles in the regulation of cell proliferation, cell adhesion, cancer metastasis, tissue differentiation and apoptosis [7,8]. In particular, sialylation of sugar chains has been suggested to be a very important process during development, cancer evolution, and progression, and sialic acid is often responsible for tumor-associated antigenicity [25-27]. Several studies in cervical cancer have been directed to analyze the level of sialic acid in patients' serum. The results showed that the level of these sugars is increased in patients with cervical cancer and this level return to normal after therapy. The level show a decline in patients who respond well to treatment and show an increase in patients with recurrence of cancer even prior to any clinical evidence [12,28]. The change in sialic acid expression in premalignant lesions has been scarcely studied. Roy and Chakraborty reported a slight elevation in benign lesions, moderate elevation in severe dysplasia and preinvasive carcinoma and higher elevation in invasive carcinoma of the cervix of this monosaccharide [13]. The present study describes the change of α2,3 and α2,6 sialic acid expression in cervical intraepithelial neoplasia. An increased expression of sialic acid was detected in both types of linkages related with the grade of cervical neoplasia in most of the studied samples. α2,3 Sialic acid expression in normal samples was detected in the basal layer, increasing distribution to the intermediate layers in LSIL. In some HSIL it was detected in all layers. α2,6 Sialic acid expression was not detected in the basal layer but was detected in the medium and superficial layers and in the same way that α2,3 sialic acid in HSIL is expressed in all layers. It is important to note that there exists a co-expression of both types of sialic acid linkages in the squamous intraepithelial lesions. The increased sialylation detected may play an important role in cellular interactions, changing the structure of the epithelium and losing definition of the different layers. The change in sialic acid expression has been correlated in most studies with a change in the expression of sialyltransferases genes [7,29,30]. In cervical cancer, an increase in mRNA levels of sialyltransferases ST3Gal III and ST6Gal I related with invasion has been detected [22]. A previous study from our group found an increase in the mRNA levels of the sialyltransferases ST6Gal I, ST3Gal III and ST3Gal IV in premalignant lesions of the cervix [23]. The results obtained in the presence study may be as a result of increased expression of certain sialyltransferases genes.

## Conclusion

Neoplastic transformation in the human cervical epithelium was accompanied by increased expression of α2,6 and α2,3 sialic acids. These results show that the change in sialylation occurs before cancer development and may play an important role in cellular transformation. The results may be useful for the development of diagnostic techniques for cervical intraepithelial neoplasia using quantitative methods such as flow cytometry.

## Competing interests

The authors declare that they have no competing interests.

## Authors' contributions

DLM processed the samples, analyzed data, and reviewed literature. GSL analyzed data and reviewed manuscript. JRL performed literature review, drafted the majority of manuscript. EZ analyzed data and reviewed manuscript. VVR conceived, designed and coordinated the study and participated in data analysis and drafted the manuscript. All authors have read and approved the final manuscript.
